# Lipocalin 2 regulates expression of MHC class I molecules in *Mycobacterium tuberculosis-*infected dendritic cells via ROS production

**DOI:** 10.1186/s13578-021-00686-2

**Published:** 2021-09-25

**Authors:** Ji-Ae Choi, Soo-Na Cho, Junghwan Lee, Sang-Hun Son, Doan Tam Nguyen, Seong-Ahn Lee, Chang-Hwa Song

**Affiliations:** 1 Department of Microbiology, Department of Medical Science, College of Medicine, Chungnam National University, 266 Munhwa-ro, Jung-gu, 35015 Daejeon, South Korea; 2 Department of Medical Science, College of Medicine, Chungnam National University, 266 Munhwa-ro, Jung-gu, Daejeon, 35015 South Korea; 3 Translational Immunology Institute, Chungnam National University, 99 Daehak-ro, Yuseong-gu, 34134 Daejeon, South Korea

**Keywords:** Lipocalin 2, *Mycobacterium tuberculosis*, MHC class I, Reactive oxygen species

## Abstract

**Background:**

Iron has important roles as an essential nutrient for all life forms and as an effector of the host defense mechanism against pathogenic infection. Lipocalin 2 (LCN2), an innate immune protein, plays a crucial role in iron transport and inflammation. In the present study, we examined the role of LCN2 in immune cells during *Mycobacterium tuberculosis* (Mtb) infection.

**Results:**

We found that infection with Mtb H37Ra induced LCN2 production in bone marrow-derived dendritic cells (BMDCs). Notably, expression of MHC class I molecules was significantly reduced in LCN2^−/−^ BMDCs during Mtb infection. The reduced expression of MHC class I molecules was associated with the formation of a peptide loading complex through LCN2-mediated reactive oxygen species production. The reduced expression of MHC class I molecules affected CD8^+^ T-cell proliferation in LCN2^−/−^ mice infected with Mtb. The difference in the population of CD8^+^ effector T cells might affect the survival of intracellular Mtb. We also found a reduction of the inflammation response, including serum inflammatory cytokines and lung inflammation in LCN2^−/−^ mice, compared with wild-type mice, during Mtb infection.

**Conclusions:**

These data suggest that LCN2-mediated reactive oxygen species affects expression of MHC class I molecules in BMDCs, leading to lower levels of CD8^+^ effector T-cell proliferation during mycobacterial infection.

**Supplementary Information:**

The online version contains supplementary material available at 10.1186/s13578-021-00686-2.

## Introduction

Tuberculosis (TB) caused by *Mycobacterium tuberculosis* (Mtb) has long been one of the greatest causes of death worldwide [1]. The spread of TB has been exacerbated by the emergence of multidrug-resistant TB and extensively drug-resistant TB [1]. Hence, a full understanding of the Mtb-host interaction is needed to establish a strategy for the treatment of TB.

Iron is a crucial nutrient for cell metabolism and plays key roles in DNA synthesis and cellular respiration, which are both important for cell growth and proliferation [2]. Iron is also essential for many pathogenic bacteria, which take up iron via the production of siderophores [3]. During infection, pathogenic bacteria compete with the host for iron acquisition [4]. Administration of iron into culture media has been shown to enhance the growth of Mtb in human macrophages [5]. An Mtb mutant lacking the mbtB gene, which encodes an enzyme in the biosynthetic pathway of the siderophore mycobactin, exhibits a significantly slower growth rate compared to wild-type (WT) Mtb in human THP-1 macrophages [6]. In addition, excess amounts of iron enhance Mtb growth in the lungs and spleen of BALB/C mice [7]. Thus, the regulation of iron availability is important in the pathogenesis of TB.

Lipocalin 2 (LCN2), a member of the lipocalin protein family, is a transporter of small molecules such as steroids, lipopolysaccharides, iron, and fatty acids in the circulation [8]. LCN2 binds to microbial siderophores such as mycobactin, carboxymycobactin, bacillibactin, and enterobactin [9–11]. The expression of LCN2 in alveolar macrophages and alveolar epithelia is crucial for controlling intracellular Mtb growth [12, 13]. LCN2 promotes neutrophil recruitment via regulation of chemokine production in macrophages during Mtb infection [14]. In addition, LCN2-deficient neutrophils exhibit impaired ability to phagocytose intra- and extracellular bacteria (*Listeria monocytogenes*, *Candida albicans*, and *Staphylococcus aureus*) [15].

Mtb is able to escape from the phagosome to the cytosol by disrupting the phagosomal membrane [16]. Previous reports suggest that the regulation of major histocompatibility complex (MHC) class I-mediated antigen presentation is important for controlling intracellular Mtb growth [17, 18]. Endogenous peptides are loaded onto MHC class I molecules via peptide loading complex (PLC) components such as calreticulin (CRT), ERp57, tapasin, and protein disulfide isomerase (PDI), which stabilize the conformation of MHC class I molecules [19, 20]. Endoplasmic reticulum aminopeptidase (ERAP) associated with antigen processing trims the peptides before they are loaded onto MHC class I molecules [21, 22]. Antigen loading onto MHC class I molecules can be regulated by PLC components; however, little is known regarding the precise role of PLC in Mtb infection.

Alveolar macrophages and dendritic cells (DCs) are major immune cell types involved in the initial phagocytic process activated in response to Mtb infection [23]. DCs are important because they act as a bridge between innate and adaptive immunity in Mtb infection [23]. Because antigen presentation through MHC class I molecules is important for CD8^+^ T-cell activation, we investigated the role of LCN2 in the function of MHC class I-mediated CD8^+^ T cells. CD8^+^ T cells perform cytolytic functions to kill Mtb-infected cells via perforin, granzymes, and granulysin or apoptosis; they also produce critical cytokines such as interleukin (IL)-2, interferon (IFN)-γ, and tumor necrosis factor (TNF)-α, which act in response to Mtb infection [24]. However, the function of LCN2 in DCs during Mtb infection is unknown. The present study investigated the role of LCN2 in the regulation of intracellular Mtb growth via bone marrow-derived dendritic cell (BMDC) antigen presentation.

## Results

### Mtb-induced LCN2 is associated with expression of MHC class I molecules in BMDCs

**Fig. 1:**
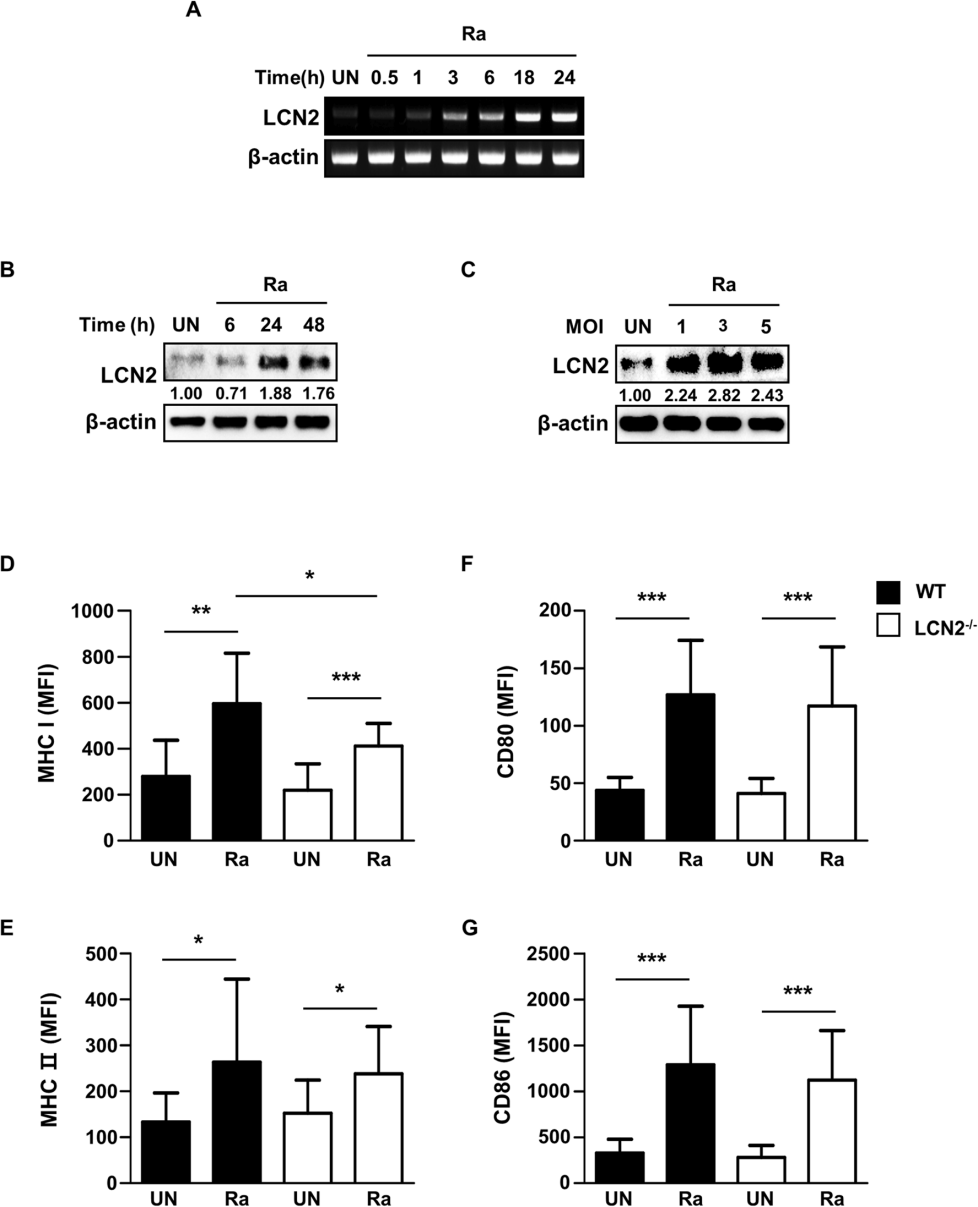
Mtb-induced LCN2 is associated with MHC class I molecule expression in BMDCs. **A**, **B** BMDCs derived from WT mice were infected with Mtb at an MOI of 1 for the indicated time periods. BMDCs were screened for the induction of LCN2 mRNA and protein during Mtb infection. **C** BMDCs derived from WT mice were infected with Mtb at a range MOI of 1 to 5 for 48 h and then screed for LCN2 protein. Western blot bands corresponding to each protein were quantified, and the intensity of each target protein was normalized to the intensity of the β-actin loading control. The normalized ratio of the unstimulated control (UN) was set as 1.0 to compare target protein abundance in different samples. The normalized ratio is shown at the bottom of the blots. A representative blot from three independent experiments is shown. **D**–**G** BMDCs derived from WT and LCN2^−/−^ mice were infected with Mtb at an MOI of 1 for 48 h. Expression levels of CD80, CD86, and MHC classes I and II molecules were analyzed on CD11c^+^-gated BMDCs by flow cytometry using specific antibodies. Data are shown as mean ± SD (n = 12). Statistically significant differences are determined by Mann–Whitney test (non‐parametric unpaired two-tailed t-test). *p < 0.05, **p < 0.01, and ***p < 0.001

To determine whether Mtb infection induces LCN2 expression in DCs, we measured the induction of LCN2 in Mtb-infected BMDCs. As shown in Fig. 1A, LCN2 mRNA levels in BMDCs increased in a time-dependent manner in response to Mtb infection. Production of the LCN2 protein also increased after 24 h and increased in an MOI-dependent manner in BMDCs infected with Mtb H37Ra (Fig. 1B, C and Additional file 1: Figure S1). Next, we evaluated the expression levels of surface markers (e.g., CD80, CD86, and MHC class I and II molecules) of WT and LCN2^−/−^ BMDCs infected with Mtb for 48 h (Fig. 1D–G). The expression levels of MHC class I molecules were significantly reduced in LCN2^−/−^ BMDCs, compared to WT BMDCs, during Mtb infection (Fig. 1D). The expression levels of other costimulatory molecules did not significantly differ between WT and LCN2^−/−^ BMDCs (Fig. 1F, G). These findings suggested that LCN2 is involved in the expression of MHC class I molecules in BMDCs during Mtb infection.

### LCN2-induced ROS are involved in expression of MHC class I molecules in Mtb-infected BMDCs

**Fig. 2:**
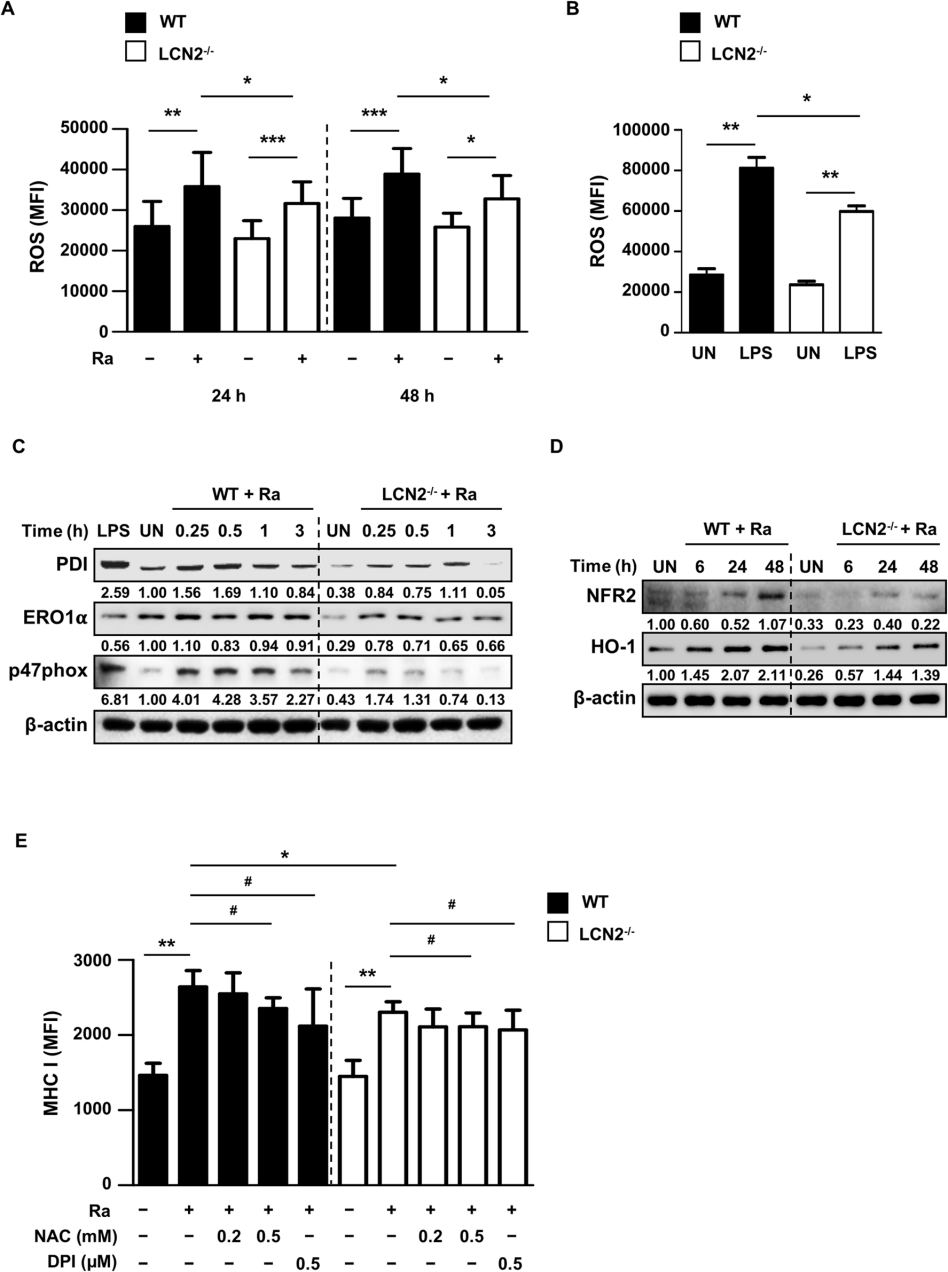
LCN2-mediated ROS production is involved in MHC class I molecule expression during Mtb infection. **A** BMDCs derived from WT and LCN2^−/−^ mice were infected with Mtb at an MOI of 1 for the indicated time periods, then stained using ROS-ID Total ROS detection kit (1 µM, 37 °C, 30 min). Data are shown as mean ± SD (n = 12). **B** BMDCs derived from WT and LCN2^−/−^ mice were stimulated with LPS (100 ng/ml) for 24 h, then stained using ROS-ID Total ROS detection kit (1 µM, 37 °C, 30 min). Data are shown as mean ± SD (n = 5). Intracellular ROS level was detected by flow cytometry in CD11c^+^-gated BMDCs. **C**, **D** BMDCs derived from WT and LCN2^−/−^ mice were infected with Mtb at an MOI of 1 for the indicated time periods. Western blotting was performed using antibodies against PDI, ERO1α, p47phox, NRF2 and HO-1. LPS (1 µg/ml, 1 h) were used as positive controls. Western blot bands corresponding to each protein were quantified, and the intensity of each target protein was normalized to the intensity of the β-actin loading control. The normalized ratio of the WT unstimulated control (UN) was set as 1.0 to compare target protein abundance in different samples. The normalized ratio is shown at the bottom of the blots. A representative blot from three independent experiments is shown. **E** BMDCs derived from WT and LCN2^−/−^ mice were preincubated with NAC (0.2 or 0.5 mM) or DPI (0.5 µM) for 30 min, then infected with Mtb at an MOI of 1 for 48 h. The expression of MHC class I molecules was analyzed by flow cytometry on CD11c^+^‐gated BMDCs. Data are shown as mean ± SD (n = 6). Statistically significant differences were determined by Mann–Whitney test (non‐parametric unpaired two-tailed t-test; *) or Wilcoxon signed-rank test (non‐parametric paired two-tailed t-test; ^#^). *p < 0.05, **p < 0.01, and ***p < 0.001; ^#^*p* < 0.05, ^##^*p* < 0.01, and ^###^*p* < 0.001

LCN2 is associated with iron homeostasis and intracellular iron levels are related to ROS production [25, 26]. We monitored whether LCN2 affects the induction of intracellular ROS during Mtb infection. The level of Mtb-induced intracellular ROS was significantly elevated in WT BMDCs, compared to LCN2^−/−^ BMDCs (Fig. 2A). Similarly, LPS-induced ROS production was significantly reduced in LCN2^−/−^ BMDCs (Fig. 2B). The levels of PDI, ERO1α, and p47phox, which are responsible for the generation of ROS, were also elevated in WT BMDCs, compared to LCN2^−/−^ BMDCs, after Mtb infection (Fig. 2C and Additional file 1: Figure S2A). Additionally, the levels of antioxidant proteins (e.g., NRF2 and HO-1) were lower in LCN2^−/−^ BMDCs than in WT BMDCs after Mtb infection (Fig. 2D and Additional file 1: Figure S2B). Next, we examined whether intracellular ROS were involved in the expression of MHC class I molecules in BMDCs during Mtb infection. As expected, Mtb-induced expression of MHC class I molecules was significantly reduced by treatment with 0.5 mM N-acetyl cysteine (NAC; ROS scavenger) or 0.5 µM diphenyleneiodonium (DPI; NOX inhibitor) at 48 h in both WT and LCN2^−/−^ BMDCs (Fig. 2E).

**Fig. 3:**
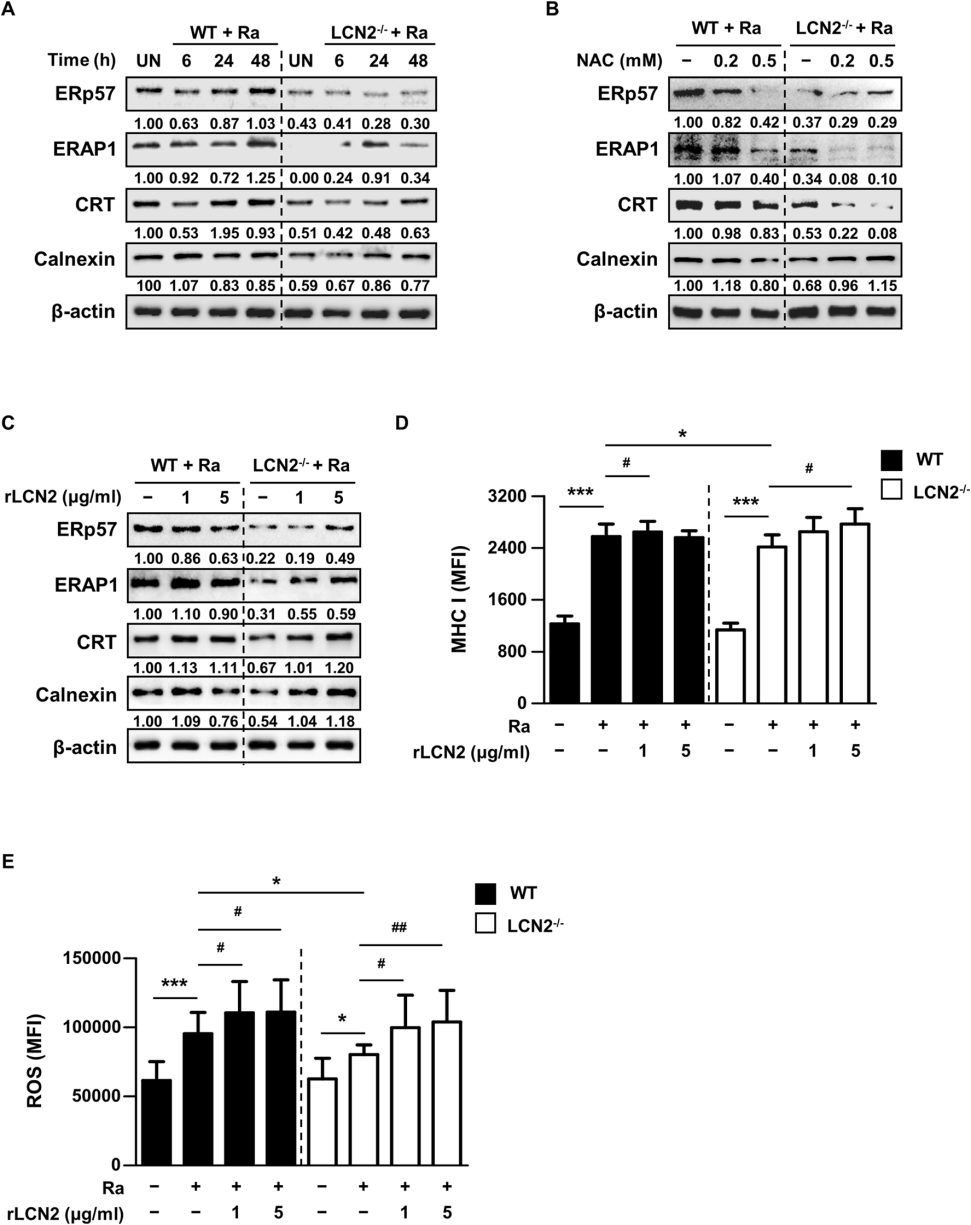
Effects of LCN2-mediated ROS on formation of the PLC in BMDCs. **A** BMDCs derived from WT and LCN2^−/−^ mice were infected with Mtb at an MOI of 1 for the indicated time periods. **B** BMDCs derived from WT and LCN2^−/−^ mice were preincubated with NAC (0.2 or 0.5 mM) for 30 min, then infected with Mtb at an MOI of 1 for 48 h. Western blotting was performed using antibodies against CRT, ERp57, ERAP1, and calnexin. **C**–**E** BMDCs derived from WT and LCN2^−/−^ mice were infected with Mtb at an MOI of 1, then incubated with recombinant LCN2 (1 or 5 µg/ml) for 48 h. **C** Western blotting was performed using antibodies against CRT, ERp57, ERAP1, and calnexin. Western blot bands corresponding to each protein were quantified, and the intensity of each target protein was normalized to the intensity of the β-actin loading control. The normalized ratio of the WT control (WT unstimulated control (UN) or Ra-only infected WT control) was set as 1.0 to compare target protein abundance in different samples. The normalized ratio is shown at the bottom of the blots. A representative blot from three independent experiments is shown. **D** The expression of MHC class I molecules was analyzed by flow cytometry on CD11c^+^-gated BMDCs. Data are shown as mean ± SD (n = 8). **E** Levels of intracellular ROS were determined by flow cytometry using ROS-ID Total ROS detection kit (1 µM, 37 °C, 30 min) in CD11c^+^‐gated BMDCs. Data are shown as mean ± SD (n = 10). Statistically significant differences were determined by Mann–Whitney test (non‐parametric unpaired two-tailed t-test; *) or Wilcoxon signed-rank test (non‐parametric paired two-tailed t-test; ^#^). *p < 0.05, **p < 0.01, and ***p < 0.001; ^#^*p* < 0.05, ^##^*p* < 0.01, and ^###^*p* < 0.001

Next, we hypothesized that LCN2-mediated ROS played a role in MHC class I antigen presentation through PLC. To confirm this hypothesis, we investigated the expression levels of PLC-related molecules in Mtb-infected BMDCs. As shown in Fig. 3A, the levels of PLC-related proteins (CRT, ERp57, calnexin, and ERAP1) were reduced in Mtb-infected LCN2^−/−^ BMDCs, compared to WT BMDCs. The levels of PLC-related proteins were suppressed in WT BMDCs pretreated with NAC. Specifically, the levels of ERp57, ERAP1 and CRT were markedly reduced in NAC-treated WT BMDCs (Fig. 3B). The expression of PLC-related protein was also decreased in LCN2^−/−^ BMDCs pretreated with NAC (Fig. 3B).

We examined the levels of ERp57, ERAP1, CRT, calnexin, and MHC class I molecules in LCN2^−/−^ BMDCs after the addition of recombinant LCN2 protein (rLCN2). Notably, the addition of rLCN2 recovered the reduced levels of PLC-related proteins in LCN2-deficient BMDCs (Fig. 3C). The production of PLC-related molecules increased slightly in WT BMDCs following the addition of 1 µg/mL rLCN2 (Fig. 3C). Expression of MHC class I molecules in LCN2^−/−^ BMDCs was significantly enhanced following the addition of 5 µg/mL rLCN2. Similarly, MHC I expression in WT BMDCs was significantly increased by adding 1 µg/mL rLCN2 (Fig. 3D). Interestingly, intracellular ROS levels in both WT and LCN2^−/−^ BMDCs were dose-dependently increased by rLCN2 supplementation at 48 h after Mtb infection (Fig. 3E). Our findings suggest that LCN2-mediated ROS induce expression of MHC class I molecules in BMDCs through the formation of PLC during Mtb infection.

### LCN2-mediated expression of MHC class I molecules affects the generation of effector CD8^+^ T cells during Mtb infection

**Fig. 4:**
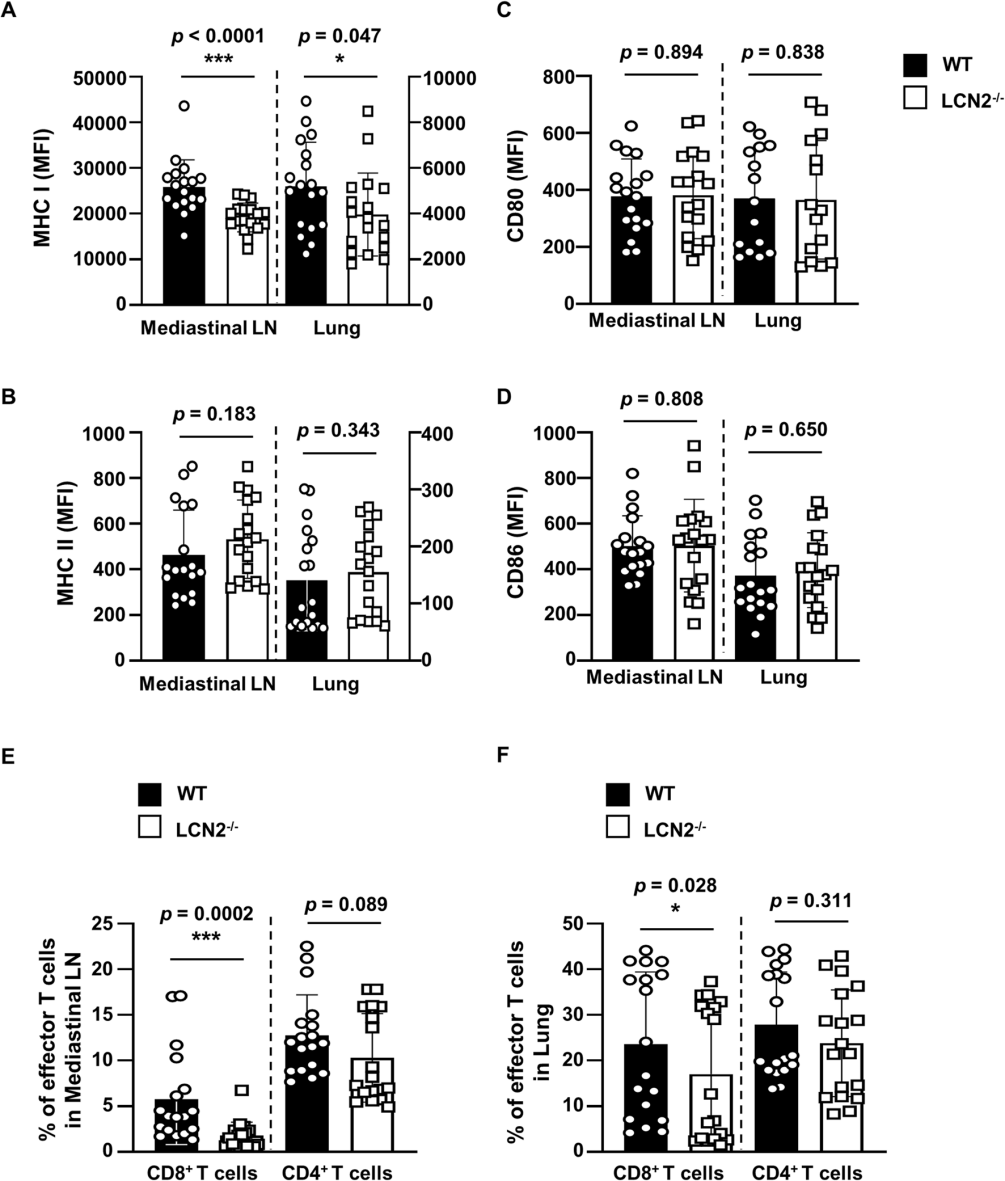
LCN2 regulates MHC class I molecule expression and CD8^+^ effector T-cell generation following intratracheal Mtb infection. WT and LCN2^−/−^ mice were intratracheally infected with Mtb (10^6^ CFU) for 10 days. **A**–**D** Expression levels of CD80, CD86, and MHC classes I and II molecules were analyzed by flow cytometry using specific antibodies in CD11c^+^ cell populations from mediastinal lymph nodes and lungs. Data are shown as mean ± SD (n = 18, 6 mice/group) and are pooled from three independent experiments. **E**, **F** Levels of effector T cells (CD62L^−^CD44^+^) were detected by flow cytometry in CD3^+^ cell populations from mediastinal lymph nodes and lungs. Data are shown as mean ± SD (n = 18, 6 mice/group) and are pooled from three independent experiments. Statistically significant differences were determined by Mann–Whitney test (non-parametric unpaired two-tailed t-test). *p < 0.05, and ***p < 0.001 LN, lymph node

To investigate the role of LCN2 in the activation of DCs during Mtb infection, we examined the expression levels of MHC class I, MHC class II, and costimulatory molecules in CD11c^+^ cell populations from the mediastinal lymph nodes and lungs of WT and LCN2^−/−^ mice after intratracheal infection with Mtb for 10 days. Among the BMDC surface markers, only expression of MHC class I molecules was significantly reduced in the mediastinal lymph nodes (*p* < 0.0001) and lung (*p* = 0.047) of LCN2^−/−^ mice, compared to WT mice (Fig. 4A). The expression levels of CD86 and MHC class II molecules were slightly elevated in the mediastinal lymph nodes and lungs of LCN2^−/−^ mice, compared to WT mice (Fig. 4B, D). The expression levels of CD80 were not different in the mediastinal lymph nodes and lungs of between WT and LCN2^−/−^ mice (Fig. 4C). Similarly, expression of MHC class I molecules was significantly reduced, but the expression level of CD80 was enhanced in the inguinal lymph nodes of LCN2^−/−^ mice after intravenous infection with Mtb for 30 days (Additional file 1: Figure S4A).

To compare the effector T-cell populations in WT and LCN2^−/−^ mice, the mice were intratracheally infected with Mtb for 10 days (Fig. 4E, F). The effector CD8^+^ T-cell population was significantly smaller in the mediastinal lymph nodes (*p* = 0.0002) and lungs (*p* = 0.028) of the LCN2^−/−^ mice, compared to WT mice. The effector CD4^+^ T-cell population was slightly reduced in the mediastinal lymph nodes (*p* = 0.089) and lungs (*p* = 0.311) of the LCN2^−/−^ mice, compared to WT mice. However, the effector CD4^+^ T-cell population did not significantly differ between WT and LCN2^−/−^ mice. The effector CD8^+^ T-cell population was significantly reduced in inguinal lymph nodes (*p* = 0.03) and spleen (*p* = 0.03) of the LCN2^−/−^ mice, compared to WT mice (Additional file 1: Figure S4B, C).

Next, we examined whether the reduced levels of total T-cells in LCN2^−/−^ mice affected the reduced effector CD8^+^ T-cell population during Mtb infection. The CD8^+^ T-cell population was slightly enhanced in the mediastinal and inguinal lymph nodes of LCN2^−/−^ mice, compared to WT mice. However, the CD3^+^, CD4^+^, and CD8^+^ T-cell populations did not significantly differ between the WT and LCN2^−/−^ mice (Additional file 1: Figure S5). In addition, rLCN2 compensation significantly increased the effector CD8^+^ T-cell population when CD3^+^ naïve T cells were co-cultured with LCN2^−/−^ BMDCs infected with Mtb (Additional file 1: Figure S6). Taken together, these results suggest that LCN2 affects the generation of effector CD8^+^ T cells by regulating the expression of MHC class I molecules in DCs during Mtb infection.

To confirm whether Mtb primed-CD8^+^ T-cells suppressed intracellular Mtb growth, CD8^+^ T- cells were isolated from the spleen of mice that had been infected with Mtb for 10 days; the cells were then co-cultured with Mtb-infected WT BMDMs. As expected, Mtb-primed CD8^+^ T-cells of WT and LCN2^−/−^ mice significantly suppressed the intracellular survival of Mtb, compared to the uninfected control. Notably, CD8^+^ T-cells from WT mice more effectively suppressed Mtb growth, compared to CD8^+^ T cells from LCN2^−/−^ mice (Additional file 1: Figure S7).

### LCN2 regulates intracellular Mtb survival

**Fig. 5:**
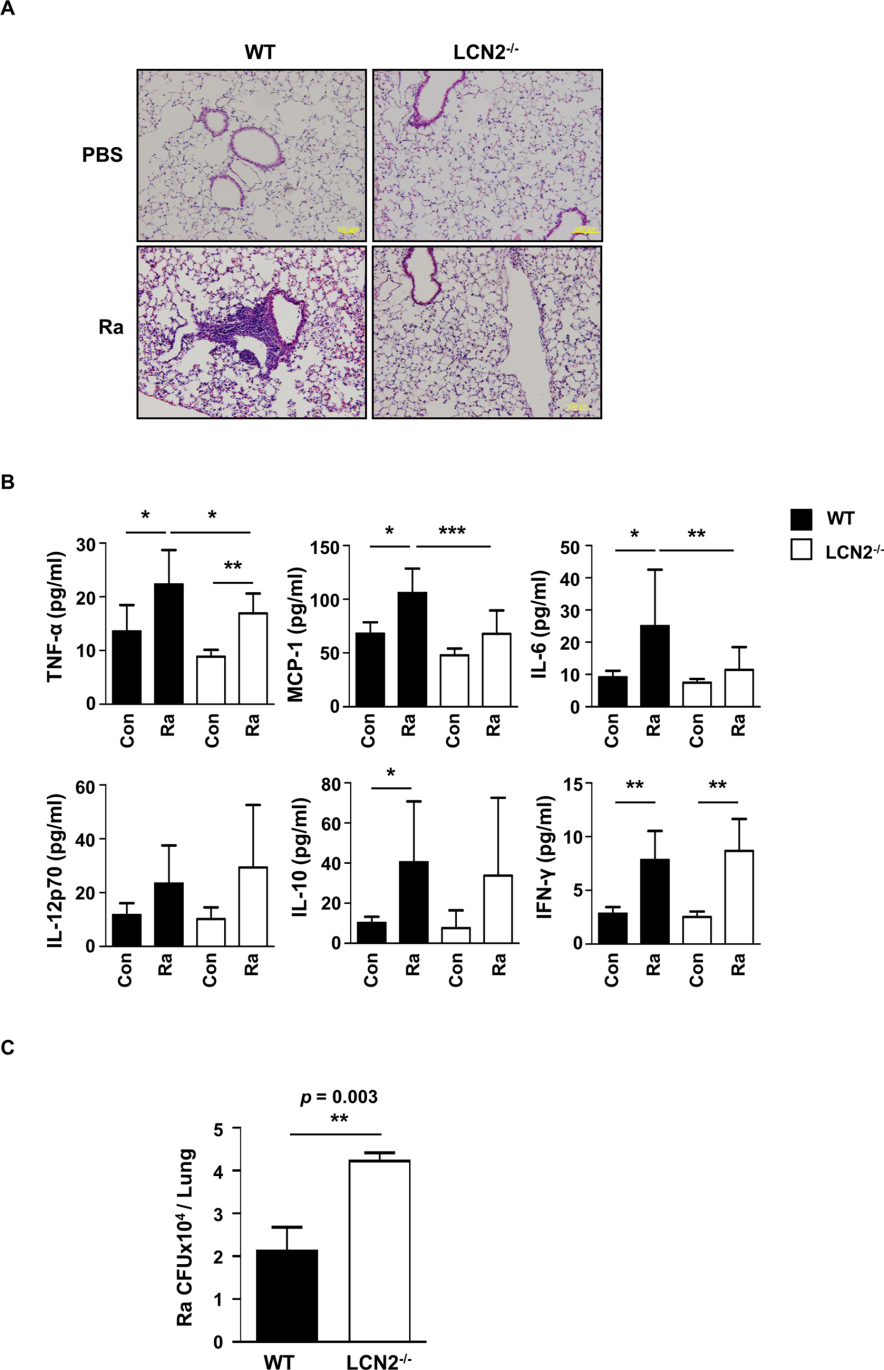
LCN2 promotes inflammation following Mtb infection. WT and LCN2^−/−^ mice were intratracheally infected with Mtb (10^6^ CFU) for 10 days. **A** Lung inflammation was determined by hematoxylin and eosin staining. Sale bars = 200 μm. **B** Levels of inflammatory cytokines in sera were determined using a CBA mouse inflammation kit by flowcytometry. Data are shown as mean ± SD (n = 9, 3 mice/group) and are pooled from three independent experiments. **C** Bacterial loads in the lungs were analyzed by CFU assays on 7H10 plates. Data are shown as mean ± SD (n = 9, 3 mice/group) and are are pooled from three independent experiments. Statistically significant differences were determined by Mann–Whitney test (non-parametric unpaired two-tailed t-test). *p < 0.05, **p < 0.01, and ***p < 0.001

Because inflammation plays a role in the regulation of Mtb growth [27], we measured the inflammation level in an *in vivo* system during Mtb infection. WT and LCN2^−/−^ mice were intratracheally infected with Mtb for 10 days, after which the histological aspects of the lungs were monitored. The data showed that inflammatory cells were slightly enhanced in the lung tissues of WT mice, compared to the LCN2^−/−^ mice (Fig. 5A and Additional file 1: Figure S8A). Inflammatory cytokines such as IL-6, TNF-α, and MCP-1 were significantly reduced in the sera of LCN2^−/−^ mice, compared to WT mice (Fig. 5B). Similarly, levels of IL-6 and MCP-1 were significantly lower in the lung homogenates of LCN2^−/−^ mice compared to in WT mice (Additional file 1: Figure S8B). These results suggested that LCN2 is closely associated with inflammation in Mtb-infected mice.

To establish the effect of LCN2 on the regulation of intracellular survival of Mtb in an *in vivo* system, we measured intracellular Mtb by CFU counts in the lungs at 10 days post-infection. Mtb survival was significantly enhanced in the lungs of LCN2^−/−^ mice, compared to WT mice (*p* = 0.003) (Fig. 5C). Similarly, intracellular Mtb was significantly enhanced in the spleen of LCN2^−/−^ mice at 30 days post-infection, compared to WT mice (*p* = 0.0001) (Additional file 1: Figure S9).

Our findings suggested that Mtb-induced LCN2 is important for the generation of CD8^+^ effector T cells in response to mycobacterial infection because it affects the induction of MHC class I expression in DCs.

## Discussion

LCN2 is known to regulate growth of bacteria (e.g., *Escherichia coli* and *Salmonella typhimurium*) through iron depletion [28, 29]. LCN2 has been shown to restrict the growth of Mtb by limiting replication through iron sequestration in macrophages and alveolar epithelial cells [12, 13]. In the present study, LCN2 in DCs was found to play a critical role in antigen presentation processing during Mtb infection.

Intracellular iron is responsible for the generation of ROS and the hydroxyl radical (HO⋅) via the Fenton reaction [30]. The findings of a recent study suggested that the expression of MHC class I molecules was reduced in an intracellular iron-limiting environment [31]. Here, we showed that the production of PLC components and ROS was reduced in LCN2^−/−^ BMDCs during Mtb infection. Among the PLC components, the levels of ERAP1 and calnexin were strongly suppressed by treatment with NAC, a ROS scavenger. Calnexin promotes the initial folding of MHC class I heavy chains through direct binding to the disulfide bond prior to dimerization with β2-m [32]. These results suggest that reduction of endoplasmic reticulum (ER) stress might affect PLC formation; ER stress induces CRT/calnexin production and regulates the posttranscriptional level of the ERAP1 gene [33, 34]. The results of some studies have suggested that optimal assembly of the MHC class I molecule via PLC components is essential in the host defense against Mtb infection [17, 18]. The reduced levels of intracellular iron may have a suppressive effect on PLC formation, potentially leading to the downregulation of MHC class I molecule expression in LCN2^−/−^ BMDCs. Our results suggest that ROS generation by LCN2 is an important factor affecting MHC class I molecule expression via PLC formation during Mtb infection. However, the regulation of MHC class I molecule expression is highly complex and involves multiple factors, and some factors related to MHC class I molecule expression still need to be investigated.

We showed that the levels of MHC class I molecule expression in CD11c^+^ cells were lower in the mediastinal lymph nodes of LCN2^−/−^ mice, compared to WT mice, during Mtb infection. The CD8^+^ effector T-cell populations were also significantly smaller in the mediastinal lymph nodes and lungs of the LCN2^−/−^ mice, compared to WT mice. The small population of CD8^+^ effector T-cells in the absence of LCN2 was presumably due to impaired antigen presentation capacity, based on the reduced expression of MHC class I molecules in CD11c^+^ cells in the mediastinal lymph nodes. Therefore, the reduced population of CD8^+^ effector T-cells might contribute to the enhanced intracellular survival of Mtb in the lungs of LCN2^−/−^ mice, compared to WT mice.

The levels of proinflammatory cytokines (e.g., TNF-α, IL-6, and MCP-1) were reduced in the sera of LCN2^−/−^ mice after Mtb infection. Our findings are consistent with the results of other studies, which showed that LCN2 promotes inflammation during mycobacterial infections [13, 14, 35]. MCP-1 contributes to the organization of granulomatous inflammation through recruitment and accumulation of macrophages and T lymphocytes in the lungs following early Mtb infection [27]. TNF-α enhances the ability of macrophages to phagocytose and kill mycobacteria [36, 37]. In addition, TNF-α promotes apoptosis of Mtb-infected alveolar macrophages and plays a role in the formation and maintenance of granulomas [38, 39]. IL-6 is critically important for regulating intracellular survival of Mtb [40]. Thus, reduced proinflammatory cytokine production in LCN2^−/−^ mice might constitute a favorable environment for Mtb survival.

Finally, we observed elevated numbers of intracellular Mtb in the lungs and spleen of LCN2^−/−^ mice, compared to WT mice, during Mtb infection. Iron-related immune modulatory mechanisms have been suggested to reduce iron levels and promote the reduction of IFN-γ receptors; they may also modulate the expression of MHC class I molecules [31, 41]. Furthermore, LCN2 might affect the polarization of M1 macrophages or their clearance by enhancing the phagocytic/autophagic process [42, 43]. The results of our previous study suggested that M1 macrophages were better able to control intracellular Mtb, compared to M2 macrophages [44]. In summary, our findings indicate a new crucial protective role for LCN2 in CD8^+^ effector T-cell activation via regulation of MHC class I molecule expression in BMDCs, in response to mycobacterial infection. Therefore, regulation of LCN2 production might represent an important therapeutic approach for future treatment of tuberculosis.

## Conclusions

In this study, we found that LCN2-mediated ROS generation affects MHC class I molecule expression via PLC formation during Mtb infection. LCN2-mediated expression of MHC class I molecules in DCs regulates intracellular mycobacterial growth by affecting the effector CD8^+^ T-cell population. LCN2 plays a protective role in the suppression of intracellular mycobacteria. An Mtb H37Ra infection model was used, because the gene content and sequence of the Mtb H37Ra genome is highly similar to that of Mtb H37Rv. Taken together, our findings suggest that LCN2 functions as an MHC class 1 modulator during mycobacterial infection.

## Materials and methods

### Cell cultures

Murine BMDCs were generated, cultured, and purified as previously described [45]. BMDCs were cultured at 37 °C in the presence of 5% CO_2_ in RPMI 1640 media (Welgene Co., Daegu, South Korea) supplemented with 100 unit/ml penicillin/streptomycin (Lonza, Walkersville, MD, USA), 10% fetal bovine serum (Gibco, Waltham, MA, USA), 20 ng/ml GM-CSF (CreaGene; Geyonggi-do, South Korea), and 5 ng/mL IL-4 (CreaGene) for 7 days. Murine bone marrow-derived macrophages (BMDMs) were isolated from femurs and tibias of C57BL/6 mice (6–9 weeks old) and differentiated by growth for 3–5 days in Dulbecco’s modified Eagle medium containing M-CSF (25 ng/mL; R&D Systems, Minneapolis, MN, USA).

### In vivo model

C57BL/6 WT and LCN2^−/−^ mice (male, 8–9 weeks of age) were used in this study and housed under specific pathogen-free conditions. WT mice were purchased from Samtako (Geyonggi-do, South Korea). LCN2^−/−^ mice were kindly provided by Dr. Kiyoshi Mori (Kyoto University, Kyoto, Japan) and Dr. Shizuo Akira (Osaka University, Osaka, Japan). Mice were infected intratracheally with Mtb strain H37Ra [10^6^ colony-forming units (CFU)] in 50 µl phosphate-buffered saline (PBS) for 10 days. Samples from the lungs, mediastinal lymph nodes, lung homogenates and blood sera were collected at 10 days after Mtb infection. Other mice were intravenously infected with Mtb strain H37Ra (10^6^ CFU) in 150 µl PBS for 30 days. Samples from the inguinal lymph nodes and spleen were collected at 30 days after Mtb infection. These organs were used for histological analysis, detection of cell surface markers, and CFU analysis.

### Bacterial culture and CFU assay

Mtb strain H37Ra (ATCC 25177) was purchased from the American Type Culture Collection and cultured in Middlebrook 7H9 liquid medium containing 10% oleic acid, albumin, dextrose, and catalase, combined with 5% glycerol. Aliquots of the upper bacterial suspension of Mtb H37Ra were frozen at − 80 °C until use. Cells were infected with Mtb H37Ra at a multiplicity of infection (MOI) of 1 for 3 h. To remove the extracellular mycobacteria, cells were washed with PBS and incubated with fresh medium without antibiotics for an additional period. The cells were lysed in autoclaved distilled water to release intracellular bacteria. The lysates were plated separately on 7H10 agar plates and incubated at 37 °C for 21 days.

### RNA extraction and reverse transcription-polymerase chain reaction assay

Total RNA was extracted from Mtb-infected BMDCs using TRIzol reagent (Invitrogen, Carlsbad, CA, USA), in accordance with the manufacturer’s instructions; mRNA was reverse transcribed into cDNA using the Reverse Transcription Kit (ELPIS Biotech, Daejeon, South Korea). The cDNA was amplified using specific primers.

### Western blotting assay

Western blotting analysis was performed as described previously [46] using primary antibodies against LCN2 (R&D Systems), ERp57, ERAP1 (Abcam; Cambridge, MA, USA), ERO1α (Novus Biologicals; Littleton, CO, USA), HO-1 (Enzo Life Sciences; Farmingdale, NY, USA), CRT, NRF2, calnexin, p47phox, β-actin (Santa Cruz Biotechnology; Dallas, TX, USA), and PDI (Cell Signaling; Danvers, MA, USA). Goat anti-rabbit-IgG-HRP (Cell Signaling), donkey anti-goat IgG (Santa Cruz Biotechnology), and rabbit anti-mouse-IgG-HRP (Calbiochem, San Diego, CA, USA) were used as secondary antibodies. The blots were quantified using an Alliance Mini 4 M (UVItec, Cambridge, UK). Western blot bands corresponding to each protein were quantified, and the intensity of each target protein was normalized to the intensity of the β-actin loading control. The normalized ratio of the control was set as 1.0 to compare target protein abundance in different sample. The normalized ratios of target proteins were used to compare target protein abundance in different samples. The normalized ratio is shown at the bottom of the blots. The normalized intensity values of three different experiments are plotted as mean ± standard deviation (SD).

### Reagents

Tunicamycin, NAC, and diphenyleneiodonium were purchased from Calbiochem. Lipopolysaccharide (LPS) was purchased from Sigma (St. Louis, MO, USA). These reagents were dissolved in dimethyl sulfoxide (Sigma) or distilled water.

### Reactive oxygen species (ROS) analysis

To measure intracellular ROS levels, cells were stained using ROS-ID Total ROS detection kit (Enzo Life Sciences; Farmingdale, NY, USA) for 30 min at 37 °C. After they had been washed with PBS, the cells were fixed with 4% paraformaldehyde (PFA). Samples were analyzed on a FACS Canto II cytometer (BD Biosciences). The data were processed using Flow Jo (Tree Star; Ashland, OR, USA).

### BMDC surface marker and effector T-cell analysis

BMDCs infected with Mtb at an MOI of 1 for 48 h, and total single cells extracted from the organs of WT and LCN2^−/−^ mice infected with Mtb, were stained with specific monoclonal antibodies (mAbs) for 20 min at 4 °C, then washed with PBS. After they had been washed, the cells were fixed with 4% PFA. To ascertain cell surface marker expression, cells were analyzed using a FACS Canto II cytometer (BD Biosciences). Data analysis was performed using Flow Jo software (Tree Star). For FACS analysis, fluorescein isothiocyanate-conjugated anti-CD80, allophycocyanin (APC)-conjugated anti-CD86, phycoerythrin (PE)-Cy7-conjugated anti-CD11c, PE-conjugated anti-MHC class I, Alexa700-conjugated anti-MHC class II, PE-conjugated anti-CD44, and PE-Cy7-conjugated anti-CD62L mAbs were purchased from eBioscience (San Diego, CA, USA). APC-conjugated anti-CD3, APC-Cy7-conjugated anti-CD4, and Alexa700-conjugated anti-CD8 mAbs were obtained from BD Biosciences (San Jose, CA, USA).

### Mouse sera and lung homogenates cytokine analysis

Mouse sera and lung homogenates were collected for cytokine profiling after Mtb infection, then kept frozen until batch analysis. The lung tissues were homogenized at 4 °C. Proinflammatory cytokines [e.g., IL-6, IL-12p70, TNF-α, IL-10, IFN-γ, and monocyte chemoattractant protein (MCP)-1] were quantified using a cytometric bead array (CBA) mouse inflammation kit (BD Biosciences) by a FACS Canto II cytometer (BD Biosciences); the data were processed using Flow Jo (Tree Star).

### In vitro T-cell cytotoxicity assay

Responder CD8^+^ T cells were isolated using a magnetic-activated cell sorting system (Miltenyi Biotec, Bergisch Gladbach, Germany) with CD8-coated magnetic microbeads from the spleen of WT and LCN2^−/−^ mice intravenously infected with Mtb for 10 days, in accordance with the manufacturer’s instructions. BMDMs (2 × 10^5^ cells/well) were infected with Mtb at an MOI of 1 for 3 h. After 24 h, the BMDMs were cocultured with CD8^+^ T cells (1 × 10^6^) at a BMDM:T cell ratio of 1:5. On day 6 of coculture, the intracellular bacteria were analyzed by CFU assays.

### Hematoxylin and eosin staining

Mouse lung tissues were infected with Mtb for 10 days; they were then collected, fixed in 10% formalin, and embedded in paraffin. Tissue sections on paraffin-embedded tissue arrays were deparaffinized and rehydrated using a graded alcohol series. The tissue sample slides were incubated with Gill’s V Hematoxylin (Muto, Tokyo, Japan) for 5 min, washed under running tap water for 5 min, decolorized with 0.3% HCl solution for 15 s, and rinsed with Scott’s Bluing solution for 7 min. The cytoplasm was stained with Eosin Y solution for 2 min; the slides were then dehydrated, cleared, and mounted. The stained samples were analyzed with an Axiophot microscope (Carl Zeiss, Jena, Germany).

### Statistical analysis

For statistical analyses, data obtained from independent experiments are presented as means ± SD. All experiments were performed independently and repeated at least three times. Statistically significant differences between the two groups were determined by Mann–Whitney test (non-parametric unpaired two-tailed t-test) or Wilcoxon signed-rank test (non‐parametric paired two-tailed t-test) using GraphPad Prism Software (version 5.0; GraphPad Software, San Diego, CA, USA). Differences with values of *p < 0.05, **p < 0.01, and ***p < 0.001 were considered statistically significant.

## Supplementary Information

**Additional file 1: Figure S1.** LCN2 is induced following Mtb infection in BMDCs. **Figure S2.** LCN2 induced ROS-related molecules in Mtb-infected BMDCs. **Figure S3.** Effects of LCN2-mediated ROS on formation of the PLC in BMDCs. **Figure S4.** LCN2 regulates MHC class I molecule expression and CD8^+^ effector T-cell generation following intravenous Mtb infection. **Figure S5.** LCN2 does not affect the total T-cell population following Mtb infection. **Figure S6.** LCN2 in BMDCs is important to regulate CD8^+^ effector T-cell generation following Mtb infection. **Figure S7.** Mtb-induced effector CD8^+^ T cells regulate intracellular Mtb. **Figure S8.** LCN2 induced ROS-related molecules in Mtb-infected BMDCs. **Figure S9.** LCN2 regulates intracellular Mtb survival.

## Data Availability

The datasets supporting the conclusions of this article are included within the article and its additional files.

## Abbreviations

Mtb: *Mycobacterium tuberculosis*
TB: Tuberculosis
ROS: Reactive oxygen species
LCN2: Lipocalin 2
PLC: Peptide loading complex
MHC: Major histocompatibility complex
ERAP: Endoplasmic reticulum aminopeptidase
PDI: Protein disulfide isomerase
CRT: Calreticulin
MOI: Multiplicity of infection
CFU: Colony-forming units
GM-CSF: Granulocyte-macrophage colony-stimulating factor
M-CSF: Macrophage colony-stimulating factor
NAC: *N*-acetyl cysteine
DPI: Diphenyleneiodonium
BMDCs: Bone marrow derived-dendritic cells
BMDMs: Bone marrow derived-macrophages
TNF: Tumor necrosis factor
IL: Interleukin
IFN: Interferon
LPS: Lipopolysaccharide
LN: Lymph node
WT: Wild-type
ER: Endoplasmic reticulum
PBS: Phosphate-buffered saline
PFA: Paraformaldehyde
APC: Allophycocyanin
PE: Phycoerythrin
mAbs: Monoclonal antibodies
MCP: Monocyte chemoattractant protein
