# Supplementary material for: Lipocalin 2 regulates expression of MHC class I molecules in Mycobacterium tuberculosis-infected dendritic cells via ROS production
Source: Cell Biosci. 2021 Sep 25;11:175. doi: 10.1186/s13578-021-00686-2 (PMC8466733; doi:10.1186/s13578-021-00686-2)
Supplement: Supplementary file 1 — Additional file 1: Figure S1. LCN2 is induced following Mtb infection in BMDCs. Figure S2. LCN2 induced ROS-related molecules in Mtb-infected BMDCs. Figure S3. Effects of LCN2-mediated ROS on formation of the PLC in BMDCs. Figure S4. LCN2 regulates MHC class I molecule expression and CD8+ effector T-cell generation following intravenous Mtb infection. Figure S5. LCN2 does not affect the total T-cell population following Mtb infection. Figure S6. LCN2 in BMDCs is important to regulate CD8+ effector T-cell generation following Mtb infection. Figure S7. Mtb-induced effector CD8+ T cells regulate intracellular Mtb. Figure S8. LCN2 induced ROS-related molecules in Mtb-infected BMDCs. Figure S9. LCN2 regulates intracellular Mtb survival. [file 13578_2021_686_MOESM1_ESM.pdf]

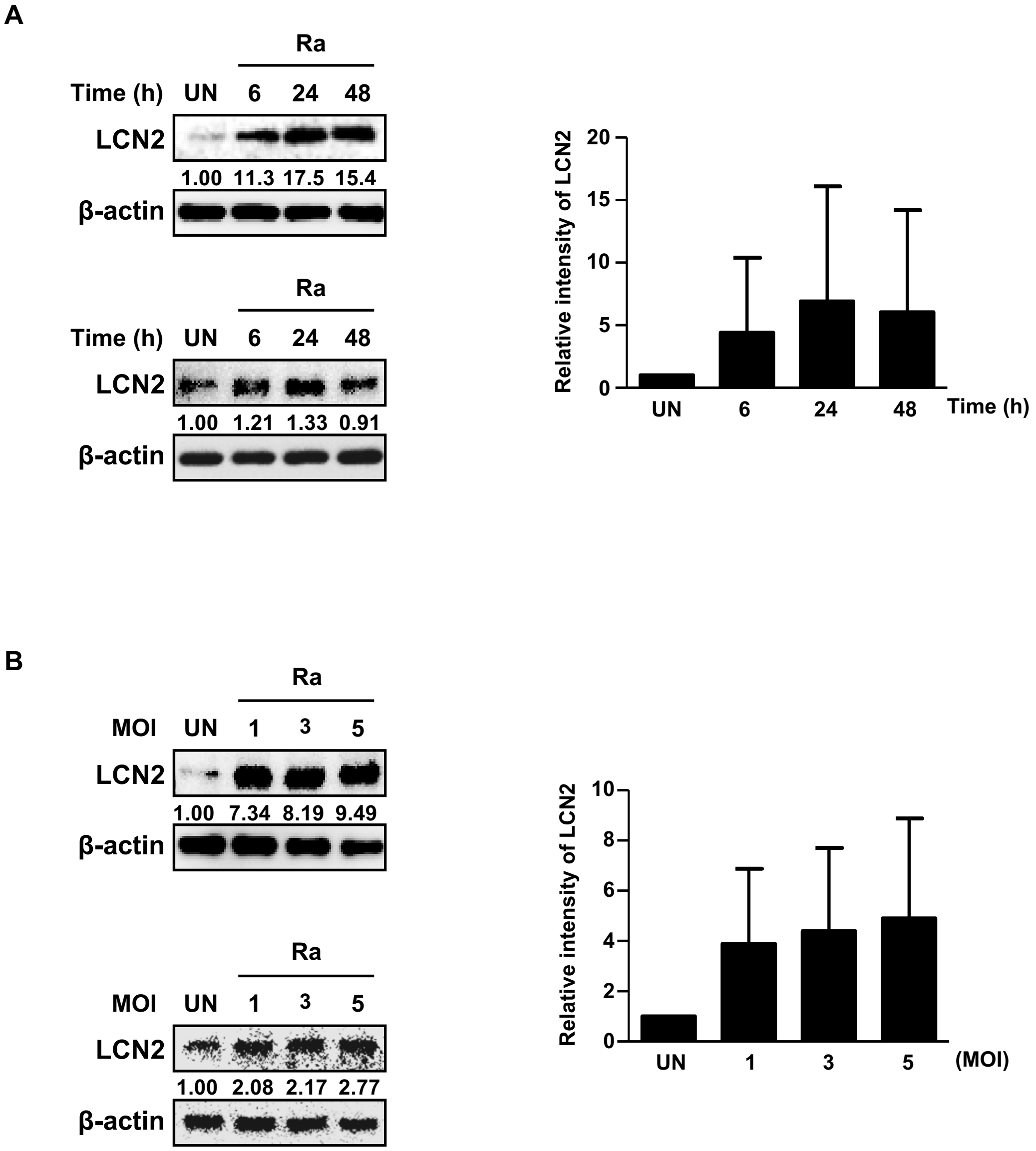


**Figure S1. LCN2 is induced following Mtb infection in BMDCs.** (A) BMDCs derived from WT mice w ere infected with Mtb at an MOI of 1 for the indicated time periods. (B) BMDCs derived from WT mice w ere infected with Mtb at a range MOI of 1 to 5 for 48 h. Western blot bands corresponding to each protein were quantified, and the intensity of each target protein was normalized to the intensity of the β-actin loading control. The normalized ratio of the unstimulated control (UN) was set as 1.0 to compare target protein abundance in different samples. The normalized ratio is shown at the bottom of the blots. The normalized intensity values of three different experiments are plotted as mean ± SD.

**Figure S2**

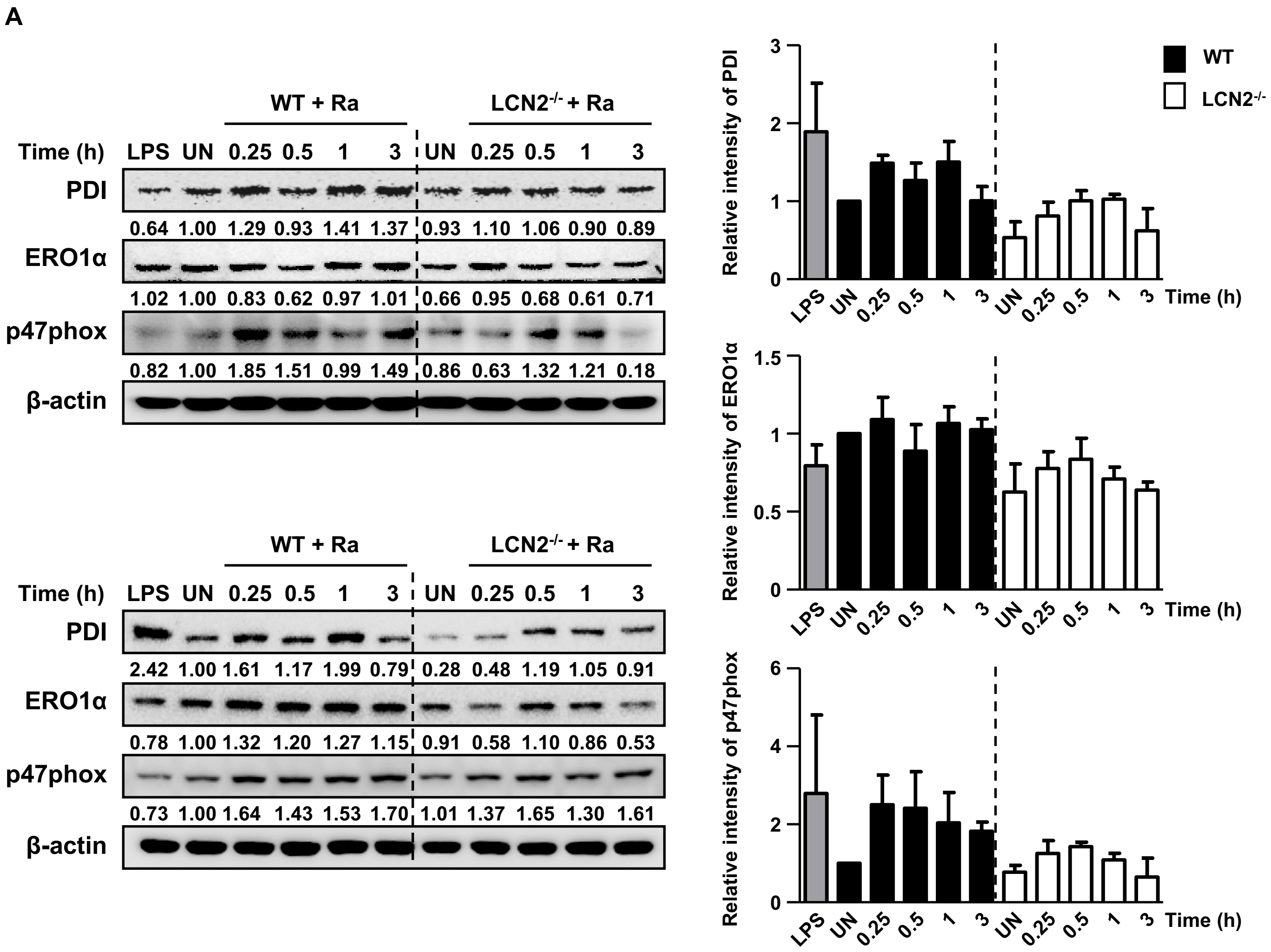


**Figure S2. LCN2 induced ROS-related molecules in Mtb-infected BMDCs.** (A, B) BMDCs derived from WT and LCN2$^{-/-}$ mice were infected with Mtb at an MOI of 1 for the indicated time periods. (A) Western blotting was performed using antibodies against PDI, ERO1α and p47phox. (B) Western blotting was performed using antibodies against NRF2 and HO-1. Western blot bands corresponding to each protein were quantified, and the intensity of each target protein was normalized to the intensity of the β-actin loading control. The normalized ratio of the unstimulated control (UN) was set as 1.0 to compare target protein abundance in different samples. The normalized ratio is shown at the bottom of the blots. The normalized intensity values of three different experiments are plotted as mean ± SD.

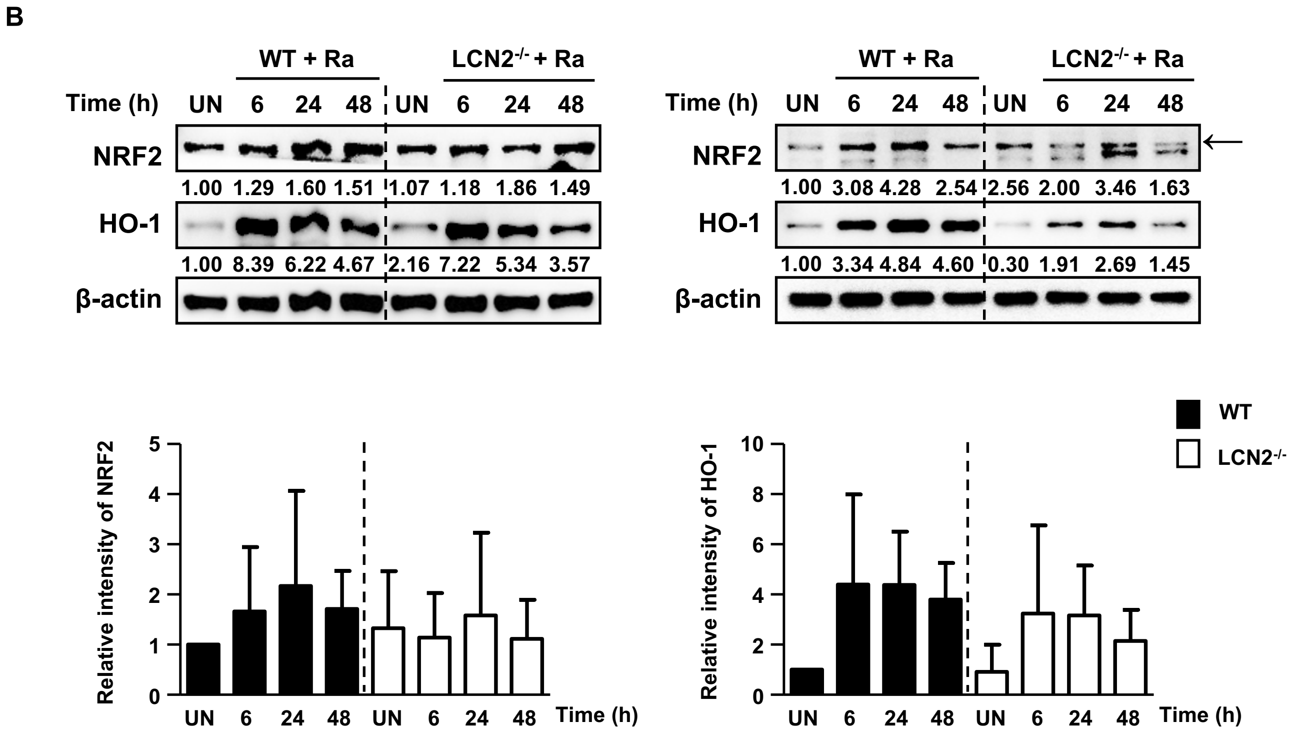
B
WT + Ra
LCN2-/- + Ra
Time (h)
UN 6 24 48 UN 6 24 48
NRF2
1.00 1.29 1.60 1.51 1.07 1.18 1.86 1.49
HO-1
1.00 8.39 6.22 4.67 2.16 7.22 5.34 3.57
β-actin
WT + Ra
LCN2-/- + Ra
Time (h)
UN 6 24 48 UN 6 24 48
NRF2
1.00 3.08 4.28 2.54 2.56 2.00 3.46 1.63
HO-1
1.00 3.34 4.84 4.60 0.30 1.91 2.69 1.45
β-actin
WT
LCN2-/-
Relative intensity of NRF2
Relative intensity of HO-1
Time (h)

**Figure S3**

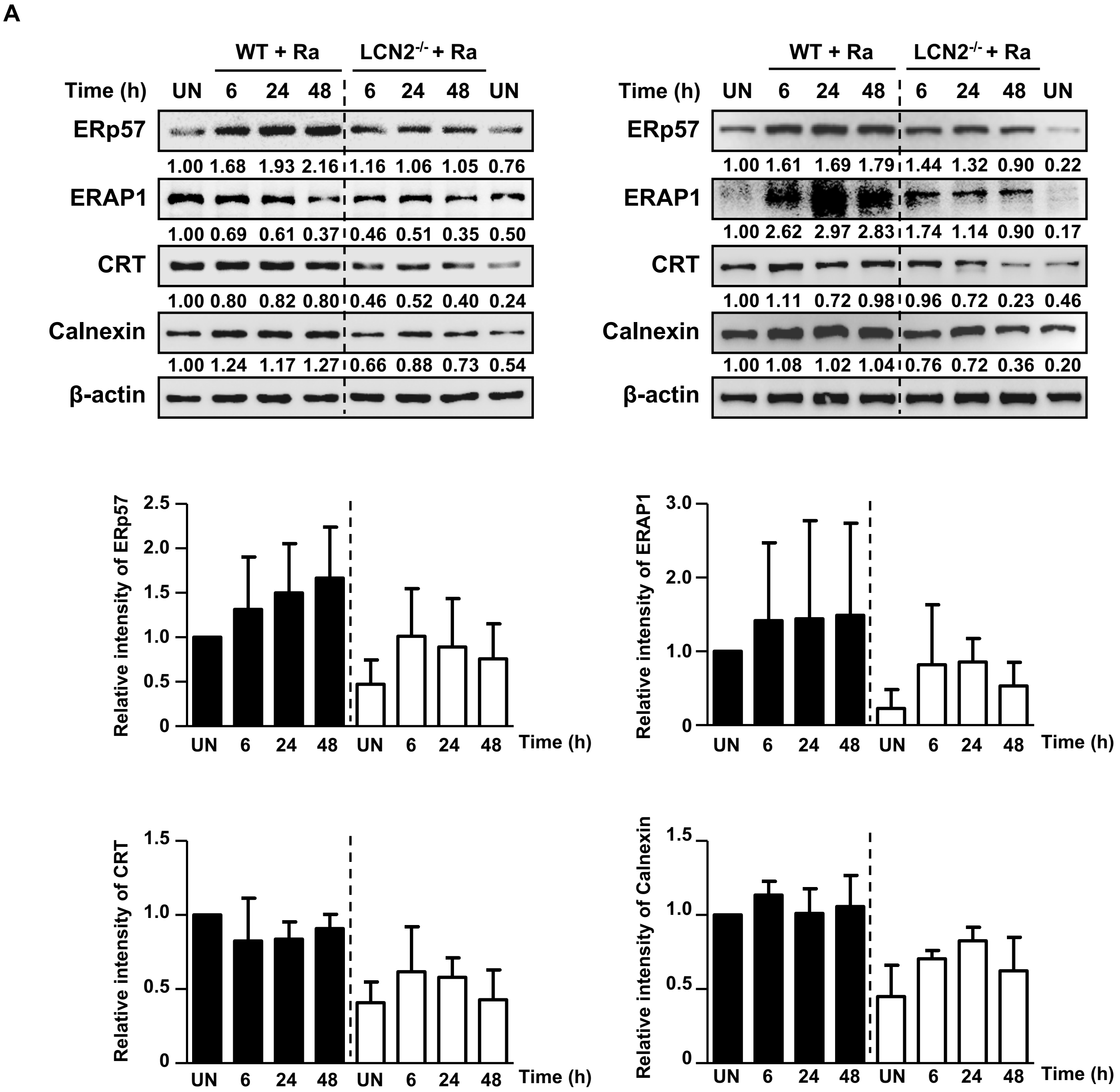


**Figure S3. Effects of LCN2-mediated ROS on formation of the PLC in BMDCs.** (A) BMDCs derived from WT and LCN2$^{-/-}$ mice were infected with Mtb at an MOI of 1 for the indicated time periods. (B) BM DCs derived from WT and LCN2-/- mice were preincubated with NAC (0.2, 0.5 mM) for 30 min, then inf ected with Mtb at an MOI of 1 for 48 h. (C) BMDCs derived from WT and LCN2-/- mice were infected wi th Mtb at an MOI of 1, then incubated with recombinant LCN2 (1, 5 µg/ml) for 48 h. Western blotting was performed using antibodies against CRT, ERp57, ERAP1, and Calnexin. Western blot bands corresponding to each protein were quantified, and the intensity of each target protein was normalized to the intensity of the β-actin loading control. The normalized ratio of the WT control (WT unstimulated control (UN) or only Ra-infected WT control) was set as 1.0 to compare target protein abundance in different samples. The normalized ratio is shown at the bottom of the blots. The normalized intensity values of three different experiments are plotted as mean ± SD.

**Figure S3**

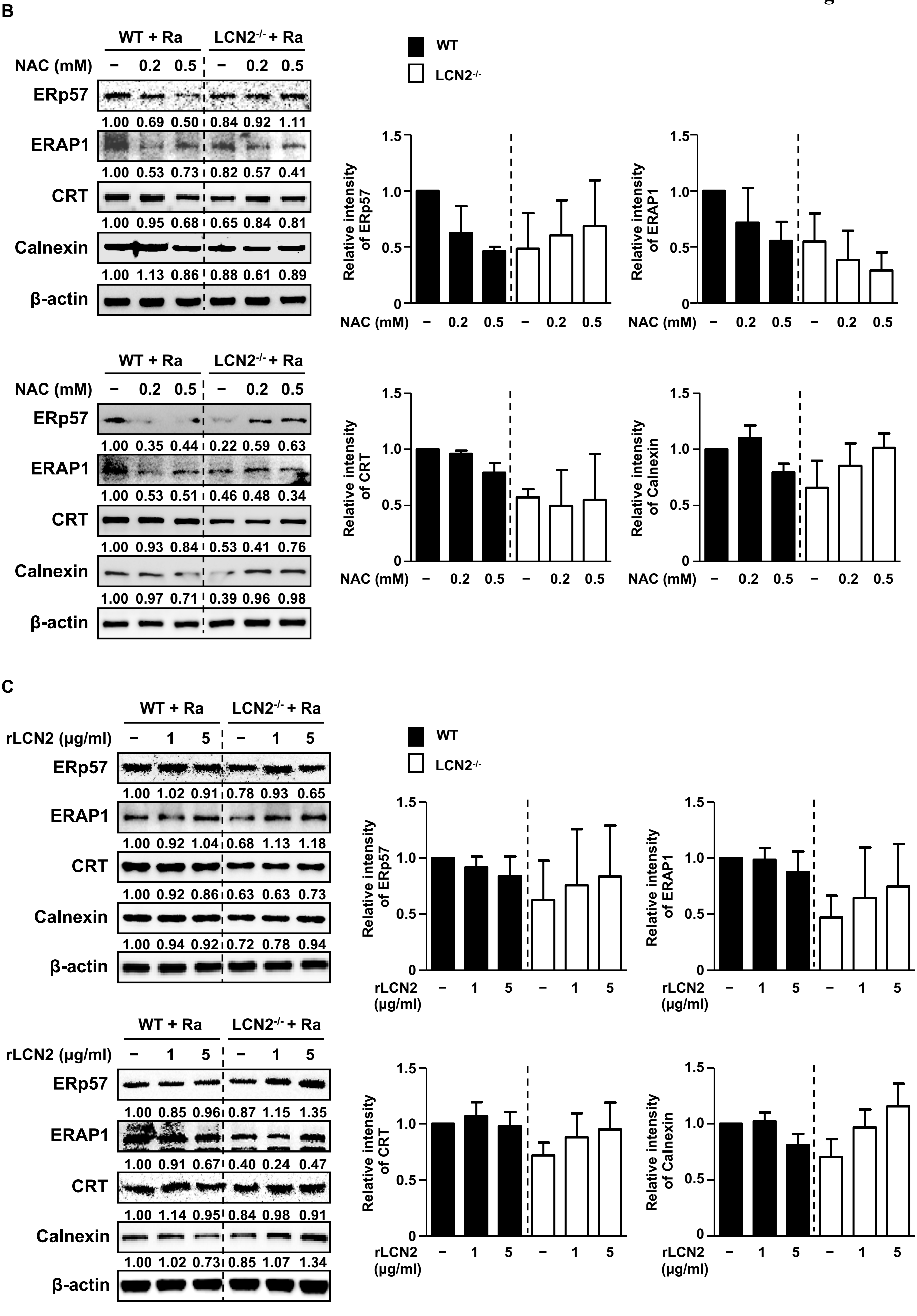
B
WT + Ra
LCN2-/- + Ra
NAC (mM) – 0.2 0.5 – 0.2 0.5
ERp57
1.00 0.69 0.50 0.84 0.92 1.11
ERAP1
1.00 0.53 0.73 0.82 0.57 0.41
CRT
1.00 0.95 0.68 0.65 0.84 0.81
Calnexin
1.00 1.13 0.86 0.88 0.61 0.89
β-actin
WT
LCN2-/-
Relative intensity of ERp57
Relative intensity of ERAP1
1.5
1.0
0.5
0
WT + Ra
LCN2-/- + Ra
NAC (mM) – 0.2 0.5 – 0.2 0.5
ERp57
1.00 0.35 0.44 0.22 0.59 0.63
ERAP1
1.00 0.53 0.51 0.46 0.48 0.34
CRT
1.00 0.93 0.84 0.53 0.41 0.76
Calnexin
1.00 0.97 0.71 0.39 0.96 0.98
β-actin
Relative intensity of CRT
Relative intensity of Calnexin
C
WT + Ra
LCN2-/- + Ra
rLCN2 (μg/ml) – 1 5 – 1 5
ERp57
1.00 1.02 0.91 0.78 0.93 0.65
ERAP1
1.00 0.92 1.04 0.68 1.13 1.18
CRT
1.00 0.92 0.86 0.63 0.63 0.73
Calnexin
1.00 0.94 0.92 0.72 0.78 0.94
β-actin
WT
LCN2-/-
Relative intensity of ERp57
Relative intensity of ERAP1
rLCN2 (μg/ml) – 1 5 – 1 5
WT + Ra
LCN2-/- + Ra
rLCN2 (μg/ml) – 1 5 – 1 5
ERp57
1.00 0.85 0.96 0.87 1.15 1.35
ERAP1
1.00 0.91 0.67 0.40 0.24 0.47
CRT
1.00 1.14 0.95 0.84 0.98 0.91
Calnexin
1.00 1.02 0.73 0.85 1.07 1.34
β-actin
Relative intensity of CRT
Relative intensity of Calnexin

**Figure S4**

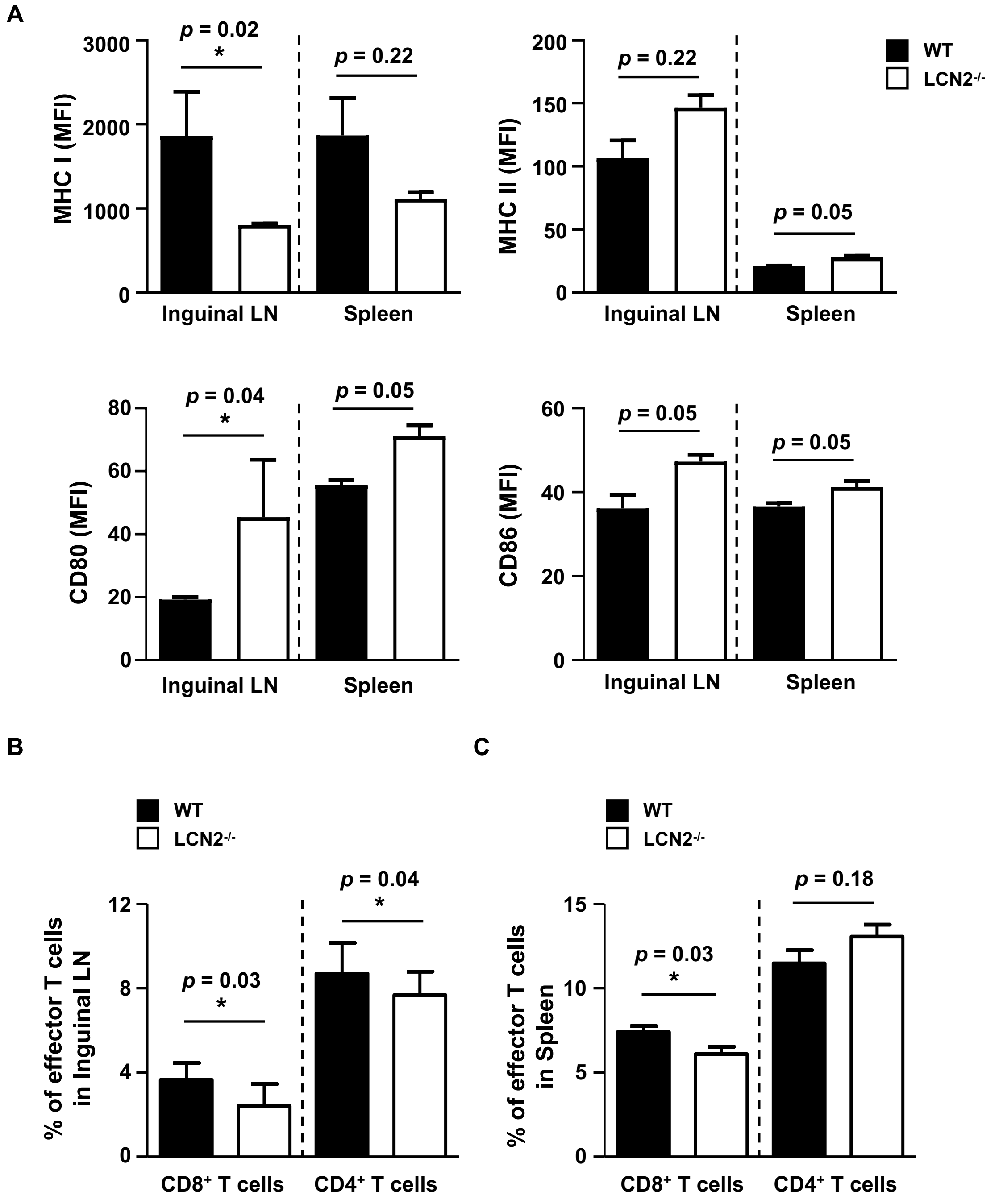


**Figure S4. LCN2 regulates MHC class I molecule expression and $CD8^+$ effector T-cell generation following intravenous Mtb infection.** WT and $LCN2^{-/-}$ mice were intravenously infected with Mtb ($10^6$ CFU) for 30 days. (A) Expression levels of CD80, CD86, and MHC classes I and II molecules were analyzed by flow cytometry in $CD11c^+$ cell populations from inguinal lymph nodes and spleen. Data are shown as mean ± SD (n = 6, 3 mice/group) and are pooled from two independent experiments. (B, C) Levels of effector T cells ($CD62L^-CD44^+$) were detected by flow cytometry in $CD3^+$ cell populations in inguinal lymph nodes and spleen. Data are shown as mean ± SD (n = 9, 3 mice/group) and are pooled from three independent experiments. Statistically significant differences are determined by with the Mann-Whitney test (non-parametric unpaired two-tailed t-test). LN, lymph node. * $p < 0.05$

**Figure S5**

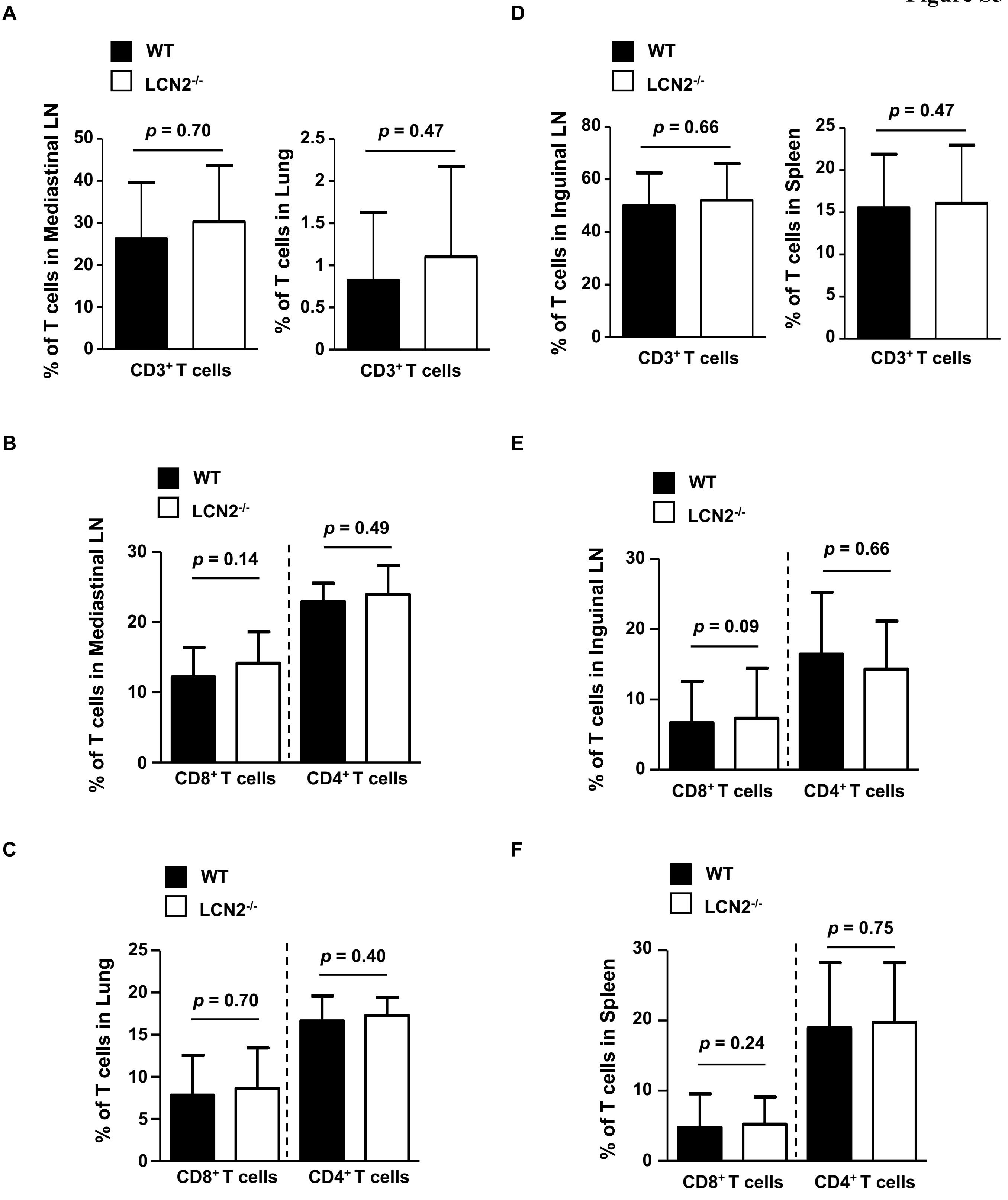


**Figure S5. LCN2 does not affect the total T-cell population following Mtb infection.** (A-C) WT and LCN2$^{-/-}$ mice were intratracheally infected with Mtb ($10^6$ CFU) for 10 days. (D-F) WT and LCN2$^{-/-}$ mice were intravenously infected with Mtb ($10^6$ CFU) for 30 days. The populations of T-cells were analyzed by flow cytometry in mediastinal lymph nodes, lungs, inguinal lymph nodes, and spleen. Data are shown as mean ± SD (n = 12, 3 mice/group) and are pooled from four independent experiments. Statistically significant differences are determined by with the Mann-Whitney test (non-parametric unpaired two-tailed t-test). LN, lymph node.

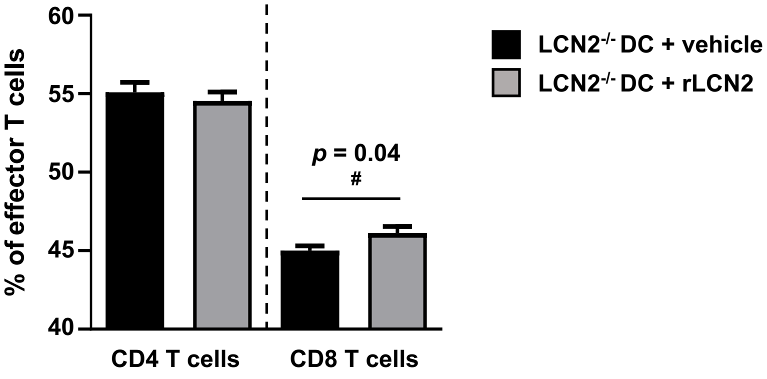


**Figure S6. LCN2 in BMDCs is important to regulate CD8$^+$ effector T-cell generation following Mtb infection.** BMDCs derived from LCN2$^{-/-}$ mice were infected with Mtb at an MOI of 1, then incubated with recombinant LCN2 (5 μg/ml) for 48 h. LCN2$^{-/-}$ BMDCs were co-cultured with CD3$^+$ naïve T cells from spleen of WT mice at a ratio of 1:5 for 6 days. Levels of effector T cells (CD62L$^-$CD44$^+$) were detected by flow cytometry in CD3$^+$ cell populations. Data are shown as mean ± SD (n = 7) and are pooled from two independent experiments. Statistically significant differences are determined by Wilcoxon signed-rank test (non-parametric paired two-tailed t-test). # $p$ <0.05

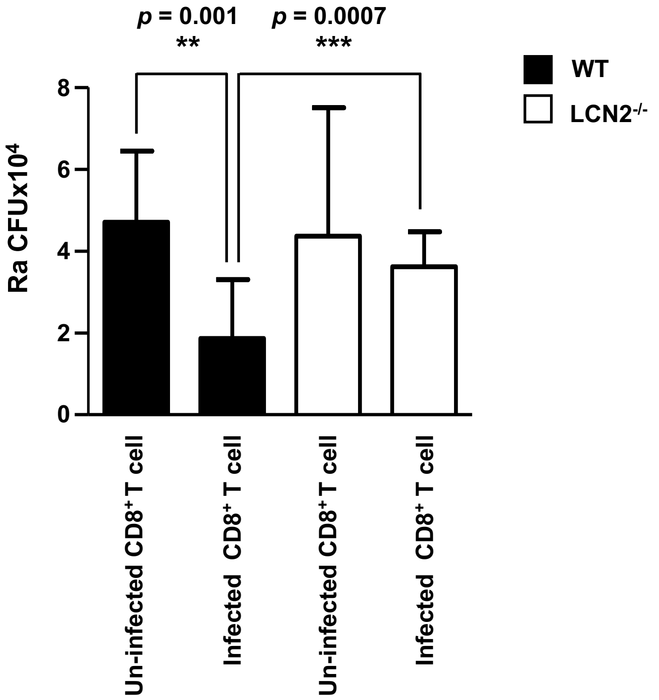


**Figure S7. Mtb-induced effector CD8$^+$ T cells regulate intracellular Mtb.** WT and LCN2$^{-/-}$ mice were intravenously infected with Mtb ($10^6$ CFU) for 10 days. CD8$^+$ T cells were isolated u sing magnetic beads conjugated with anti-CD8 from spleen of WT and LCN2$^{-/-}$ mice infected or not infected with Mtb. BMDMs derived from WT mice were infected with Mtb at an MOI of 1 f or 24 h, then co-cultured with CD8$^+$ T cells at a ratio of 1:5 for 6 days. Intracellular bacterial loa ds were analyzed by CFU assays on 7H10 plates. Data are shown as mean ± SD (n = 13). Statist ically significant differences are determined by with the Mann-Whitney test (non-parametric unp aired two-tailed t-test). ** $p < 0.01$ and *** $p < 0.001$

# Figure S8

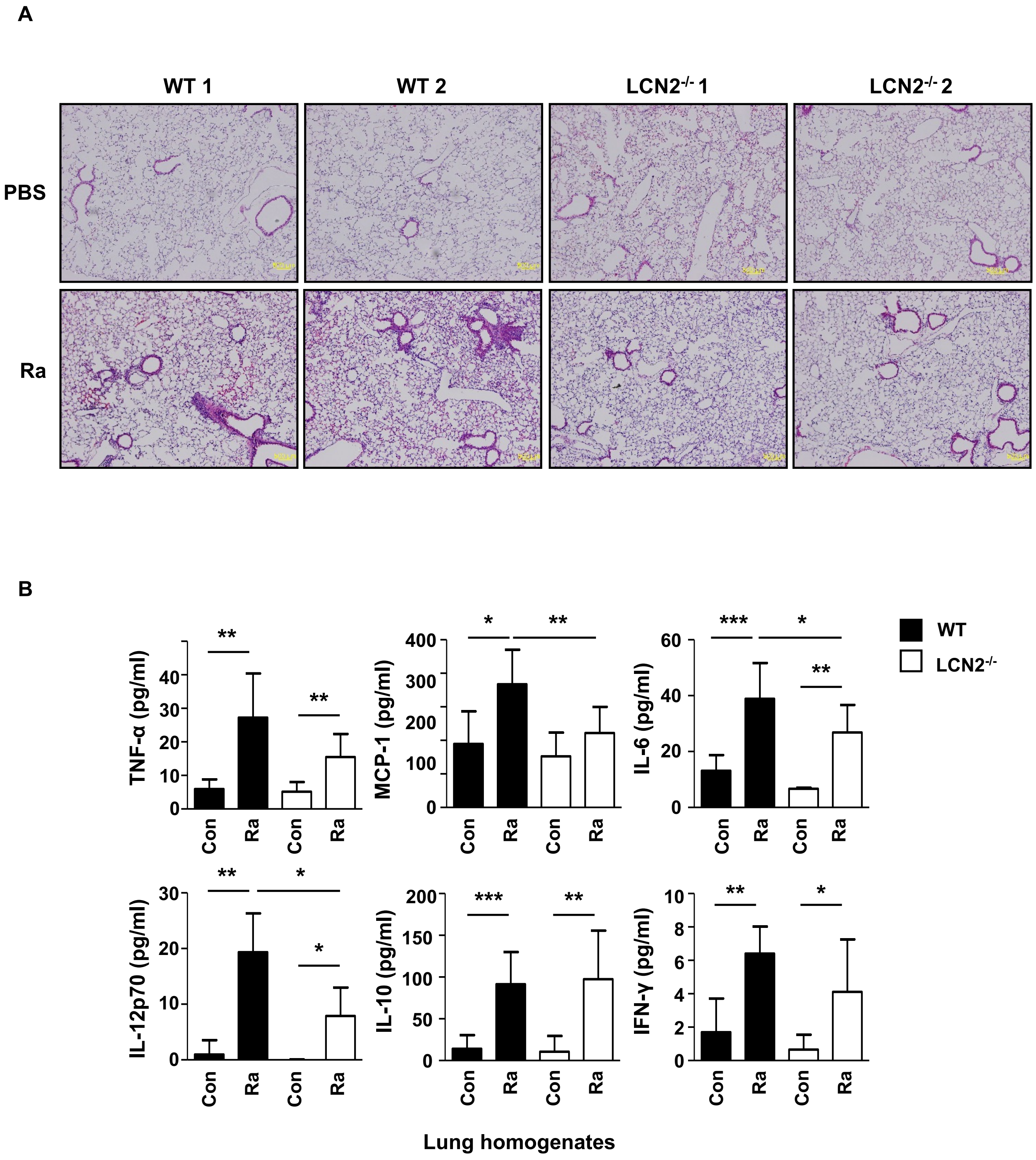


**Figure S8. LCN2 induced ROS-related molecules in Mtb-infected BMDCs.** WT and LCN2$^{-/-}$ mice wer e intratracheally infected with Mtb ($10^6$ CFU) for 10 days. (A) Lung inflammation was determined by hem atoxylin and eosin staining. Sale bars = 500 μm. (B) Levels of inflammatory cytokines in lung tissue hom ogenates were determined using a CBA mouse inflammation kit by flow cytometry. Data are shown as me an ± SD (n = 9 mice, 3 mice/group) and are pooled from three independent experiments. Statistically signi ficant differences are determined by with the Mann-Whitney test (non-parametric unpaired two-tailed t-tes t). * $p$ <0.05 , *** $p$ <0.01 and *** $p$ <0.001

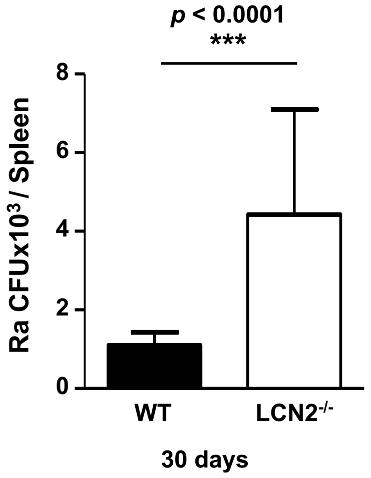


**Figure S9. LCN2 regulates intracellular Mtb survival.** WT and LCN2$^{-/-}$ mice were intravenously infected with Mtb ($10^6$ CFU) for 30 days. Intracellular bacterial loads in the spleen were analyzed b y CFU assays on 7H10 plates. Data are shown as mean ± SD (n = 12, 3 mice/group) and are pooled from four independent experiments. Statistically significant differences are determined by with the Mann-Whitney test (non-parametric unpaired two-tailed t-test). *** $p$ <0.001
